# Clinical intervals and diagnostic characteristics in a cohort of prostate cancer patients in Spain: a multicentre observational study

**DOI:** 10.1186/s12894-015-0058-x

**Published:** 2015-07-02

**Authors:** Xavier Bonfill, María José Martinez-Zapata, Robin WM Vernooij, María José Sánchez, María Morales Suárez-Varela, Javier de la Cruz, José Ignacio Emparanza, Montserrat Ferrer, José Ignacio Pijoán, Juan M. Ramos-Goñi, Joan Palou, Stefanie Schmidt, Víctor Abraira, Javier Zamora, on behalf of the EMPARO-CU study group

**Affiliations:** CIBER de Epidemiología y Salud Pública (CIBERESP), Madrid, Spain; Institute of Biomedical Research (IIB Sant Pau), Iberoamerican Cochrane Centre, Barcelona, Spain; Universitat Autònoma de Barcelona, Barcelona, Spain; Public Health and Clinical Epidemiology Service, Hospital de la Santa Creu i Sant Pau, Barcelona, Spain; Instituto de Investigación Biosanitaria de Granada, Escuela Andaluza de Salud Pública, Granada, Spain; Department of Preventive Medicine, Unit of Public Health and Environmental Care, University of Valencia, Center for Public Health Research (CSISP), Valencia, Spain; Hospital 12 de Octubre, Madrid, Spain; Clinical Epidemiology Unit, Hospital Universitario Donostia, BioDonostia, San Sebastian, Spain; IMIM (Hospital del Mar Medical Research Institute), Health Services Research Group, Barcelona, Spain; Unidad de Epidemiología Clínica y Soporte Metodológico, UICEC de BioCruces-SCReN, Barakaldo, Spain; Health Services Research on Chronic Patients Network (REDISSEC), HTA Unit of the Canary Islands Health Service (SESCS), S/C de Tenerife, La Laguna, Spain; Fundació Puigvert, Barcelona, Spain; Department of Experimental and Health Sciences, Universidad Pompeu Fabra (UPF), Barcelona, Spain; Unidad de Bioestadística Clínica, Hospital Universitario Ramón y Cajal, IRYCIS, Madrid, Spain

**Keywords:** Prostatic neoplasms, Male urogenital diseases, Multicentre study, Cohort study, Prospective study

## Abstract

**Background:**

Little is known about the healthcare process for patients with prostate cancer, mainly because hospital-based data are not routinely published. The main objective of this study was to determine the clinical characteristics of prostate cancer patients, the, diagnostic process and the factors that might influence intervals from consultation to diagnosis and from diagnosis to treatment.

**Methods:**

We conducted a multicentre, cohort study in seven hospitals in Spain. Patients’ characteristics and diagnostic and therapeutic variables were obtained from hospital records and patients’ structured interviews from October 2010 to September 2011. We used a multilevel logistic regression model to examine the association between patient care intervals and various variables influencing these intervals (age, BMI, educational level, ECOG, first specialist consultation, tumour stage, PSA, Gleason score, and presence of symptoms) and calculated the odds ratio (OR) and the interquartile range (IQR). To estimate the random inter-hospital variability, we used the median odds ratio (MOR).

**Results:**

470 patients with prostate cancer were included. Mean age was 67.8 (SD: 7.6) years and 75.4 % were physically active. Tumour size was classified as T1 in 41.0 % and as T2 in 40 % of patients, their median Gleason score was 6.0 (IQR:1.0), and 36.1 % had low risk cancer according to the D’Amico classification. The median interval between first consultation and diagnosis was 89 days (IQR:123.5) with no statistically significant variability between centres. Presence of symptoms was associated with a significantly longer interval between first consultation and diagnosis than no symptoms (OR:1.93, 95%CI 1.29–2.89). The median time between diagnosis and first treatment (therapeutic interval) was 75.0 days (IQR:78.0) and significant variability between centres was found (MOR:2.16, 95%CI 1.45–4.87). This interval was shorter in patients with a high PSA value (*p* = 0.012) and a high Gleason score (*p* = 0.026).

**Conclusions:**

Most incident prostate cancer patients in Spain are diagnosed at an early stage of an adenocarcinoma. The period to complete the diagnostic process is approximately three months whereas the therapeutic intervals vary among centres and are shorter for patients with a worse prognosis. The presence of prostatic symptoms, PSA level, and Gleason score influence all the clinical intervals differently.

## Background

Prostate cancer is the most frequently diagnosed cancer among Spanish men. With an incidence of 65.2 per 100 000 persons per year (27 853 new cases yearly 21.7 % of the total cancer in men), it is overall the second most frequent cancer in Spain [1]. Worldwide, it is the second most frequently diagnosed cancer among men (1 111 689 new cases, 15.0 % of all cancers in men) and overall the fourth most common cancer [1]. The incidence of prostate cancer has increased over the last decades, partly due to the more frequent use of diagnostic tools such as prostate-specific antigen (PSA) testing and needle biopsies in asymptomatic men [2–4]. The impact on mortality is high. Mortality rates in Spain showed a slight increase between 1980 and 1998 but have since decreased [5, 6]. In 2012, the estimated mortality associated with prostate cancer was 5481 in Spain and 307 471 worldwide, making it the third leading cause of death due to cancer for men in Spain (8.6 % of the total) and the sixth leading cause worldwide (6.6 % of the total) [1]. Furthermore, prostate cancer reduces the quality of life of patients [7, 8].

The economic burden of prostate cancer is one of the largest among malignant tumours due to the high incidence of the disease and increasing survival rates [9]. It is estimated to cost 11.85 billion USD annually in the USA [9]. Total costs for diagnosing, treating, and monitoring patients with prostate cancer for five years have been estimated to be approximately £7294.2 per patient and £92.74 million overall in the United Kingdom [10].

The Spanish Health System is funded by taxes. It offers universal coverage and is managed regionally within each of the 17 autonomous communities. Healthcare is divided into two broad areas, primary care and hospital care. Prostate cancer is generally detected in primary care centres, where patients might undergo some diagnostic tests. For confirmatory tests, however, such as a prostate biopsy, the patient is referred to a hospital for specialised healthcare. Direct access to specialised healthcare may also occur through the hospital emergency services, but this is less frequent.

Several international initiatives have been launched to obtain detailed and reliable information regarding the healthcare process for prostate cancer patients. This information includes the time intervals between first consultation to diagnosis, and first treatment. Such projects include The European Cancer Registry-based Study of Survival and Care of Cancer Patients (EUROCARE) [11], the Patient Outcome Research Teams (PORTS) [12], and the Cancer of the Prostate Strategic Urologic Research Endeavour (CAPSURE) [13]. Information can also be obtained from databases containing regional and national incidence and mortality statistics, from hospital minimum data sets, and from hospital-based cancer registries that allow a description and generic comparison of hospital healthcare [14, 15]. These sources of information, however, do not include the type of data needed to identify the diagnostic processes, therapeutic approaches, and prognostic factors in prostate cancer. Recently, one study regarding prostate cancer has been conducted in Spain, with the objective to estimate prostate cancer incidence and profile the newly-diagnosed cases using a nationwide hospital-based registry [16, 17]. However, this study fails to examine the diagnosis and therapeutic processes and possible factors influencing these time intervals. The objective of the EMPARO-CU study is to examine the clinical care process and health outcomes of patients with urologic tumours during the first year from the histopathological prostate cancer confirmation. In this paper we describe the patients’ baseline characteristics at hospital entry and the time intervals between the first consultation and diagnosis, and between diagnosis and start of treatment and possible factors influencing these intervals.

## Methods

The EMPARO-CU study is a multicentre, cohort study of bladder and prostate cancer, conducted in seven tertiary hospitals in Spain: *Fundació Puigvert-Hospital de la Santa Creu i Sant Pau* (coordinating centre) and *Hospital del Mar* in Barcelona*, Hospital Universitario 12 de Octubre* and *Hospital Universitario Ramón y Cajal* in Madrid, *Hospital Universitario Donostia* in Donostia-San Sebastián, *Hospital General Universitario de Valencia* in Valencia, and *Hospital Universitario Virgen de las Nieves* in Granada (list of participants in Appendix). The protocol was approved by the research ethics committees at each participating centre (Table 1). Patients were enrolled from October 2010 to September 2011. Consecutive patients were selected from the urologic and oncology departments at each centre. Inclusion criteria were: 1) diagnosis of prostate cancer during the study period, independently of the tumour stage; 2) diagnosis and treatment at one of the participating hospitals; and 3) agreement to participate and signed informed consent.

**Table 1:** List of ethic committees that approval the study

Hospital de la Santa Creu i Sant Pau (Barcelona)
Fundación Puigvert (Barcelona)
Hospital 12 de Octubre (Madrid)
Hospital Ramón y Cajal (Madrid)
Autonómico del País Vasco
Hospital Donosti (San Sebastián)
Hospital General Universitario de Valencia
Hospital Nuestra Señora del Mar (Barcelona)
Hospital Virgen de las Nieves (Granada)

**Fig. 1:**
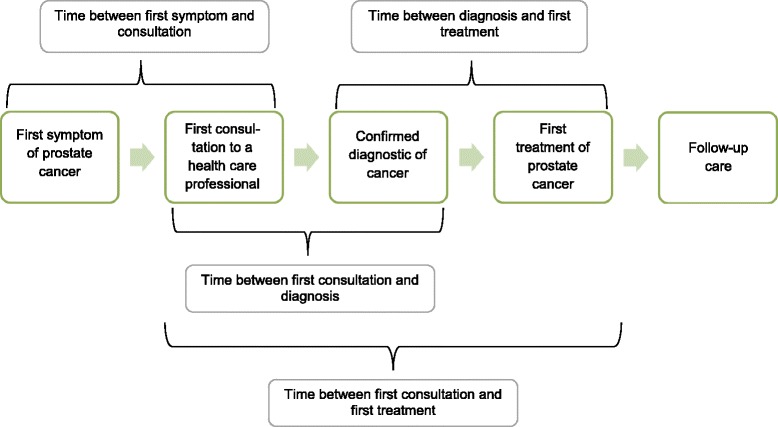
Time intervals considered in our study

The EMPARO-CU study focuses on the clinical care process and health outcomes of patients with urologic tumours. In this paper we describe the patients’ baseline characteristics at hospital entry and the intervals between the first consultation and diagnosis, and between diagnosis and start of treatment. Information regarding patient status before the diagnosis (such as symptoms at first visit) was collected retrospectively. Study data were collected from the medical records and from structured interviews with individual patients. Variables of interest were: socio-demographic data, body mass index (BMI), Charlson index, ECOG WHO score, first specialist consulted, diagnostic tests performed to establish a diagnosis of prostate cancer, pathological results of prostate biopsy [18], PSA values, total Gleason scores, clinical stages, time from first symptom to first consultation, and time from first consultation to primary diagnosis and first treatment (Fig. 1). Time from first symptom to first consultation was defined as the date on which the patients experienced the first symptoms related to prostate cancer. The date of first consultation was considered the date on which the patient first consulted a healthcare professional for the symptoms that led to prostate cancer screening. For asymptomatic patients, the first consultation was considered the date on which the physician performed prostate screening. We considered the first histological confirmation as the confirmatory diagnosis of the disease. The reference date to calculate intervals was the date of biopsy that confirmed the histological diagnosis of prostate cancer. The time interval between the first consultation and biopsy was considered the diagnostic interval. The time between the biopsy and first treatment was considered the therapeutic interval.

Categorical variables are described using relative frequency, and continuous variables are described using mean and standard deviation (SD) or median and interquartile range (IQR) for skewed distribution variables. The frequency of missing values is reported for each variable.

The association between time variables and potential predictors was assessed using multilevel (patients at first level and hospitals at second level) logistic regression models. The variables included as potential predictors in both models were age, BMI, education level, ECOG WHO score, specialist at first consultation, primary tumour clinical stage, PSA value, Gleason score, and presence of prostate cancer symptoms. Continuous time variables were transformed into dichotomous variables. In agreement with previous studies, cut-offs chosen were an interval of 100 days between first consultation and diagnosis and 30 days between diagnosis and treatment [19, 20]. These intervals were based on recommendations about optimal diagnostic and therapeutic intervals [21, 22]. We first fitted an empty model that considered only the random effect of the hospital on the variability of the two outcomes investigated. We then fitted univariate models with each potential predictor. The final model was fitted through a backward selection procedure based on Wald tests results. Both the empty model and the final multilevel models were estimated by maximum likelihood based on Gaussian quadrature points [23].

To estimate the random inter-hospital variability, we used the intra-cluster correlation coefficient (ICC) and the median odds ratio (MOR). The ICC indicates the fraction of the total outcome variability that is attributable to the area level (in our case, hospital level) and provides a measure of the within-hospital homogeneity. A lower ICC indicates a lower likelihood of patients’ sharing hospital experiences. However, because the ICC can be difficult to interpret because of binary outcomes, the partition of variance between different levels does not have the intuitive interpretation of the linear model. We therefore also calculated the MOR, defined as the median value of the odds ratio between the hospital at highest risk (longest time interval) and the hospital at lowest risk when randomly picking out two hospitals. The MOR can be conceptualised as the increased risk (in median) that a patient would have if moved to a hospital with a higher risk [24]. The measure of fixed effect was the odds ratio (OR) with 95 % confidence intervals. A *p*-value lower than 0.05 was considered statistically significant for all statistical analyses. Data analyses were performed using SPSS statistical software, version 20.0 (SPSS INC., Chicago, IL, USA) and Stata v12 (StataCorp. 2011. Stata Statistical Software: Release 12. College Station, TX: StataCorp LP).

## Results

Of the 502 patients recruited, 32 were excluded because they did not meet the inclusion criteria. The study group was therefore composed of 470 patients. Mean age was 67.8 years (SD:7.6), 337 (71.9 %) had completed at least primary studies, and 347 (73.8 %) were retired (Table 2). The mean BMI was 28.1 (SD:4.5) and 354 (75.4 %) had no physical limitations according to the ECOG WHO performance status. The Charlson comorbidity index was between one and three for 451 participants (96.2 %). Prostate screening was performed in primary care settings for 355 of the 470 patients (75.5 %), and in hospital settings for 86 of these participants (18.3 %). In 53.4 % of patients, the disease was identified during a routine visit or during consultation for another cause because no symptoms or only discomfort caused by prostate cancer had been noted. The median PSA value for the patients without symptoms was 7.2 (IQR: 6.9). From the total group, 36.2 % were symptomatic; 48.1 % of these patients had lower urinary tract symptoms such as increased frequency of urination (16.3 %), and 7.6 % had symptoms related to the tumour. The time from the first symptom to first consultation was between one month and one year for 50.8 % of participants. The clinical stage of the primary tumour was T1a-c in 41.0 % cases and T2 a-c in 40 % cases; 2.3 % had regional lymph nodes (N1) and 2.3 % had distant metastases (M1). The median PSA value was 7.6 (IQR: 7.8) ng/mL and the total Gleason score was between two and six for 55.6 % of participants. According to the D’Amico classification, 36.1 % of patients had low-risk cancer and 39.4 % had high-risk cancer (Table 2).

**Table 2:** Characteristics of prostate cancer patients

Variables	*N* = 470
	n (%)
Mean age ± SD	67.8 ± 7.6
Missing (%):	2.3
Mean BMI ± SD	28.1 ± 4.5
Missing (%)	3.8
Working status	
Active	83 (17.7)
Sick leave	15 (3.1)
Retired	347 (73.8)
Unemployed	17 (3.6)
Other	4 (0.9)
Missing	4 (0.9)
Education (%)	
No education	47 (10.2)
Incomplete primary education	80 (17.2)
Primary education	109 (23.5)
Graduate school	99 (21.3)
Upper secondary studies	62 (13.4)
University	67 (14.5)
Missing	6 (1.2)
Setting first consultation (%)	
Primary care	355 (75.5)
Hospital	86 (18.5)
Other	23 (5.0)
Missing	6 (1.2)
Symptoms (%)	
No symptoms or discomfort	251 (53.4)
One or more symptoms	170 (36.2)
Missing	49 (10.4)
Start of first symptoms including patients with discomfort (%)	
Since one month	55 (11.7)
Between one month and one year	242 (51.4)
Later than a year	56 (12.0)
No symptoms	68 (14.5)
Missing	49 (10.4)
ECOG WHO (%)	
Fully active:	356 (75.7)
Restricted or worse:	105 (22.3)
Missing:	9 (2.0)
Charlson index (%)	
1:	358 (75.8)
2:	68 (14.4)
3:	26 (5.5)
4:	13 (2.8)
≥5:	5 (1.5)
Median PSA (ng/mL) ± IQR	7.6 ± 7.8
Missing (%):	3.4
Total Gleason (%)	
2-6:	262 (55.6)
7:	127 (26.9)
>7:	74 (15.6)
Missing:	7 (1.9)
Median total Gleason ± IQR	6.0 ± 1.0
Primary tumour clinical stage (T) (%)	
Tx:	1 (0.2)
T1a-c	194 (41.0)
T2a-c:	189 (40.1)
T3a-b :	74 (15.7)
T4:	8 (1.7)
Missing:	4 (1.3)
Regional lymph nodes clinical stage (N) (%)	
Nx-N0:	460 (97.7)
N1:	10 (2.3)
Missing:	0 (0.0)
Distance metastasis clinical stage (M) (%)	
Mx-M0:	460 (97.9)
M1a-c:	10 (2.1)
Missing:	0 (0.0)
D’Amico Classification (%)	
Low risk:	170 (36.1)
Medium risk:	115 (24.5)
High risk:	185 (39.4)
Missing:	0 (0.0)
Median time between first consultation and diagnosis in days ± IQR	89.0 ± 123.5
Missing (%):	4.0
Median time between diagnosis and first treatment in days ± IQR	75.0 ± 78.0
Missing (%):	3.2
Median time between first consultation and first treatment in days ± IQR	176.0 ± 151.0
Missing (%):	6.6

All patients had a prostate biopsy and 82.9 % underwent a prostate ultrasound study. A renal ultrasonography was performed in 23.8 % of patients and a bladder ultrasonography in 22.5 % (Table 3). Table 4 shows the patients’ characteristics for each participating hospital. The median diagnostic interval was 89.0 days (IQR: 123.5). No statistically significant differences were found between hospitals for this interval (MOR: 1.00). Patients with one or more symptoms had an OR of 1.93 (95 % CI 1.29–2.89, *P* = 0.001) of having an interval between first consultation and diagnosis of more than 100 days (Table 5). No significant differences were found for groups of patients differing in age, BMI, education level, ECOG WHO score, the specialist at first consultation, primary tumour stage, PSA, or total Gleason scores.

**Table 3:** Diagnostic variables of prostate cancer patients

Diagnostic test	*N* = 470
	n (%)
Ultrasound (%)	
Prostate ultrasound:	390 (82.9)
Renal ultrasound:	112 (23.8)
Bladder ultrasound:	106 (22.5)
Puncture (%)	
Biopsy:	464 (98.7)
Aspiration:	32 (6.8)
Scintigraphy (%)	63 (13.4)
Nuclear magnetic resonance (%)	
Abdominal:	66 (14.0)
Thoracic:	1 (0.2)
Cranial:	1 (0.2)
CT scan (%)	
Abdominal:	30 (6.4)
Abdominothoracic:	33 (7.0)
Toracic:	14 (3.0)
Cranial:	3 (0.6)

**Table 4:** Characteristics of prostate cancer patients by hospitals

Centres	A (*n* = 48)	B (*n* = 91)	C (*n* = 37)	D (*n* = 78)	E (*n* = 112)	F (*n* = 33)	G (*n* = 75)
Mean age ± SD	72.6 ± 6.1	66.9 ± 7.7	67.3 ± 5.6	67.8 ± 6.7	67.1 ± 7.8	66.2 ± 6.9	67.6 ± 9.2
Missing (%):	0.0	2.2	0.0	5.1	0.0	6.1	4.1
Mean BMI ± SD	28.2 ± 5.7	27.3 ± 3.5	27.6 ± 3.8	28.8 ± 6.4	28.9 ± 4.1	27.6 ± 4.3	27.2 ± 2.9
Missing (%):	0.0	4.4	0.0	3.8	0.9	21.2	4.1
Median PSA (ng/mL) ± IQR	10.4 ± 15.5	5.7 ± 3.4	6.7 ± 3.7	7.8 ± 7.7	8.6 ± 12.2	7.0 ± 3.8	8.7 ± 8.2
Missing (%):	2.1	8.8	0.0	6.4	0.0	3.0	1.4
ECOG WHO (%)							
Fully active:	72.9	84.6	91.9	71.8	69.6	63.6	75.3
Restricted or worse:	25.0	15.4	8.1	23.1	30.4	33.3	17.8
Missing:	2.1	0.0	0.0	5.1	0.0	3.0	6.8
Primary tumour clinical stage (T) (%)							
Tx:	0.0	0.0	0.0	1.3	1.3	0.0	0.0
T1a-c	45.8	45.1	16.2	37.2	37.2	43.8	41.1
T2a-c:	43.8	39.6	56.8	37.2	37.2	33.0	19.3
T3a-b :	6.3	14.3	21.6	20.5	20.5	22.3	6.8
T4:	4.1	1.0	0.0	3.8	3.8	0.9	1.4
Missing:	0.0	0.0	5.4	0.0	0.0	0.0	1.4
Regional lymph nodes clinical stage (N) (%)							
Nx-N0:	100.0	98.9	100.0	92.3	100.0	100.0	97.3
N1:	0.0	1.1	0.0	7.7	0.0	0.0	2.7
Missing:	0.0	0.0	0.0	0.0	0.0	0.0	0.0
Distance metastasis clinical stage (M) (%)							
Mx-M0:	95.8	100.0	100.0	93.6	98.2	100.0	98.6
M1a-c:	4.2	0.0	0.0	6.4	1.8	0.0	1.4
Missing:	0.0	0.0	0.0	0.0	0.0	0.0	0.0
D’Amico Classification (%)							
Low risk:	14.6	50.5	54.1	32.1	39.3	36.4	21.9
Medium risk:	27.1	12.1	29.7	17.9	23.2	45.5	34.2
High risk:	58.3	37.4	16.2	50.0	37.5	18.1	42.5
Missing:	0.0	0.0	0.0	0.0	0.0	0.0	1.4
Total Gleason (%)							
2-6:	16.7	73.6	64.9	68.0	66.9	36.4	31.5
7:	29.2	19.8	29.7	17.9	18.8	51.5	43.8
>7:	54.1	6.6	2.7	10.2	14.3	9.1	19.2
Missing:	0.0	0.0	2.7	3.9	0.0	3.0	5.5
Median total Gleason ± IQR	8.0 ± 1.0	6.0 ± 1.0	6.0 ± 1.0	6.0 ± 2.0	6.0 ± 1.0	7.0 ± 1.0	7.0 ± 1.0
Median time between first consultation and diagnosis in days ± IQR	99.5 ± 139.0	79.0 ± 211.0	110.0 ± 117.0	78.5 ± 107.3	92.0 ± 99.0	133.0 ± 195.0	76.5 ± 100.0
Missing (%):	0.0	3.3	5.4	7.7	4.5	6.1	1.4
Median time between diagnosis and first treatment in days ± IQR	30.5 ± 127.5	78.0 ± 62.0	83.0 ± 72.0	104.0 ± 70.5	55.0 ± 66.5	73.5 ± 84.5	70.0 ± 95.0
Missing (%):	0.0	4.4	0.0	1.3	6.3	3.0	2.7
Median time between first consultation and first treatment in days ± IQR	164.0 ± 232.0	154.0 ± 207.0	212.0 ± 165.0	200.0 ± 130.0	166.0 ± 112.8	203.0 ± 149.8	165.0 ± 128.5
Missing (%):	0.0	6.6	5.4	9.0	8.9	9.1	4.1

**Table 5:** Time interval between first consultancy and diagnosis and potential determinant (Univariate regression)

			ICC/MOR	95 % CI MOR	*P*-value
Hospital random effect			0.00		
Empty model			1.00	1.00–1.00	1.000
	Median (days)	IQR (days)	OR >100 days	95 % CI OR	
Age					
<65 years	84.0	182.0	1		
≥65 years	91.0	105.5	0.96	0.64–1.43	0.830
BMI					
<25	102.0	163.0	1		
≥25	86.0	110.3	0.64	0.40–1.01	0.057
Education level					
Primary education or lower	90.5	108.5	1		
Graduate school or higher	86.0	139.0	1.00	0.69–1.45	0.992
ECOG WHO Score					
Fully active	87.5	126.8	1		
Restrictive or worse	95.0	129.0	1.18	0.76–1.83	0.474
Specialist first consultation					
Primary care	91.0	139.5	1		
Hospital or specialist	79.0	106.0	0.78	0.50–1.22	0.277
Primary tumour clinical stage (T)					
T1a–T1c	95.5	141.3	1		
T2a–T4	85.0	108.5	0.73	0.50–1.07	0.110
PSA value					
<10	95.0	135.5	1		
≥10	84.0	108.5	0.81	0.54–1.21	0.310
Total Gleason score					
<7	87.0	140.0	1		
≥7	91.0	111.5	0.87	0.60–1.27	0.464
Symptoms					
No symptoms or discomfort	83.0	110.0	1		
One or more symptoms	110.0	174.0	1.93	1.29–2.89	0.001

The median therapeutic interval was 75 days (IQR: 78.0) (Table 5). No statistically significant association was found between groups for this interval regarding age, BMI, education level, specialist at first consultation, or primary tumour stage (Table 6). A higher PSA value and a higher Gleason score shortened the interval between diagnosis and treatment. Patients with a PSA value higher than 10 or a total Gleason score higher than 7 had an OR of 0.5 (95 % CI 0.29–0.86, *P* = 0.012) and 0.53 (95 % CI 0.30–0.93, *P* = 0.026), respectively, to have an interval between diagnosis and treatment of more than 30 days. The MOR for the random effect of the hospital where the patient received care was 2.16 (95 % CI 1.45–4.87, *P* = 0.000).

**Table 6:** Time interval between diagnosis and first treatment and potential determinants

Characteristic	Univariate regression					Multivariate regression			
Hospital random effect			Empty model ICC/MOR	95 % CI MOR	*P*-value		FInal model ICC/MOR	95 % CI MOR	*P*-value
			0.18/2.22	1.52–4.58	0.000		0.17/2.16	1.45–4.87	0.000
	Median (days)	IQR (days)	OR >30 days	95 % CI OR		OR >30 days	95 % CI OR		
Age									
<65 years	78.0	74.0	1						
≥65 years	71.0	79.0	0.70	0.40–1.21	0.198				
BMI									
<25	75.0	76.0	1						
≥25	72.0	81.0	1.06	0.60–1.90	0.831				
Education level									
Primary education or lower	76.0	77.8	1						
Graduate school or higher	70.0	77.5	1.25	0.76–2.06	0.386				
ECOG WHO Score									
Fully active	77.0	74.8	1						
Restrictive or worse	54.0	77.0	0.55	0.32–0.95	0.033				
Specialist first consultation									
Primary care	76.0	77.0	1						
Hospital or specialist	74.0	79.5	0.86	0.48–1.56	0.621				
Primary tumour clinical stage (T)									
T1a–T1c	84.0	77.0	1						
T2a–T4	69.0	79.0	0.59	0.35–0.98	0.040				
PSA value									
<10	86.0	75.0	1						
≥10	50.0	81.0	0.41	0.24–0.68	0.001	0.50	0.29–0.86		0.012
Gleason score									
<7	85.5	68.8	1						
≥7	55.0	88.0	0.42	0.25–0.71	0.001	0.53	0.30–0.93		0.026
Symptoms									
No symptoms or discomfort	78.0	72.0	1						
One or more symptoms	73.5	80.3	0.62	0.37–1.05	0.073				

## Discussion

This multicentre cohort study aimed to describe the healthcare process in patients with prostate cancer in Spain. We focused on the characteristics of patients and tumours and we evaluated diagnosis and treatment delays in healthcare.

Our study included 470 patients diagnosed with prostate adenocarcinoma in a hospital care setting. Prostate biopsy and ultrasound were the most frequently performed diagnostic tests. The mean age of our population, the proportion of asymptomatic low risk patients and the median Gleason grade were similar to those reported in previous studies in Spain and in other countries [16, 19, 25–29]. The percentage of localised tumours in our population (81 %) was similar to that in an earlier study (89.8 %) in Spain conducted by Cozar *et al.* but considerably higher than that in the European study of Gatta *et al.* These discordant findings might be explained by differences between countries and years regarding accessibility to health services and physicians’ attitudes towards screening tests [30].

In our study, clinical symptoms were present in 36.2 % of all patients, the most common symptom being disorders of the lower urinary tract (48.1 %) and symptoms related with the tumour (7.6 %). These results are similar to those in the study of Cozar *et al.* where the frequency of lower urinary tract symptoms was 39.5 % of patients and the frequency of symptoms related to the tumour was 11.6 % [16]. In our study, the median interval between first consultation and diagnosis was 89.0 days, comparable to the 72 days in the study by Hansen et al. [31] and the 101 days reported by Torring et al. [32].

We did not find any variability in diagnostic interval between hospitals regarding age, BMI, education level, first visit with a specialist, tumour stage, PSA value, or Gleason score. However, the presence of symptoms lengthened this interval possibly because some symptoms of prostate cancer can be confused with benign prostatic hyperplasia.

Previous studies in Spain that determined the therapeutic intervals in cancer patients were generally conducted in a single hospital [33–35]. The most recently published multicentre study analysed this interval for six types of cancer, including prostate cancer [19], and found the mean therapeutic interval was longer than in our study (102.5 days (SD:71.6) vs. 80.4 days (SD:60.9)). However, we defined this interval as the time between the biopsy and the first oncological treatment, whereas the investigators in the previous study defined it as the time between the first diagnostic test of any kind and first oncological treatment. In contrast with the study by Perez *et al*. [19], our study was prospective, it had a larger number of cases, patients were from several different autonomous regions of the country, and information was obtained not only from medical records but also through patient interviews.

We observed that patients with a higher PSA value and a higher Gleason score had a shorter interval between diagnosis and first treatment than patients with lower values. Pérez et al. [19] reported similar findings in patients with advanced stages of prostate cancer. An explanation for this shorter interval could be that due to their worse prognosis, these patients usually receive hormonal therapy initially or exclusively, a treatment that is easier to administer **t**han radiotherapy, chemotherapy, or surgery [36].

Our results show a statistically significant variability between centres in relation to the therapeutic interval. The heterogeneity in intervals could be associated with the wide diversity in population characteristics, healthcare organisation and clinical policies in the different regions in Spain.

One of the main strengths of our study is that our sample of patients is a representative sample of the approximately 28.000 yearly incident prostatic cancer patients diagnosed in Spain because they were recruited from seven hospitals in five autonomous regions. In addition, the study’s prospective nature guarantees consistency and accuracy of the data collected, surpassing the common shortcomings of a retrospective collection of information. The study may have limitations, however, such as information bias. Given that it is based exclusively on information obtained in a hospital setting, outpatient factors such as those related to consultation at a primary level, could not have been taken into consideration. Nevertheless, as urologic cancer care is mainly provided in the hospital setting, in our view this limitation has little practical relevance.

## Conclusions

Most incident prostate cancer patients in Spain are diagnosed at an early stage of an adenocarcinoma. The period to complete the diagnostic process is approximately three months whereas the therapeutic intervals vary among centres and are shorter for patients with a worse prognosis. The presence of prostatic symptoms, PSA level, and Gleason score influence the clinical intervals differently.

## Appendix

### EMPARO study group

**Coordinating investigator:** Xavier Bonfill Cosp (Iberoamerican Cochrane Centre. Public Health and Clinical Epidemiology Service. Hospital de la Santa Creu i Sant Pau, IIB Sant Pau, Barcelona, Spain).

**Project manager:** Mª José Martínez Zapata (Iberoamerican Cochrane Centre. IIBSant Pau, Barcelona, Spain).

**Clinical research assistants:** Alborada Martínez (Universidad de Valencia); Enrique Morales Olivera (Escuela Andaluza de Salud Pública, Granada, Spain); Esther Canovas, Laura Muñoz, Gemma Mas, René Acosta, Ekaterina Popova (Iberoamerican Cochrane Centre. IIBSant Pau, Barcelona, Spain); Irma Ospina (Hospital 12 de Octubre, Madrid, Spain); Mª José Velázquez (Hospital Donostia, Donostia, Spain); Tamara Ruiz Merlo (Hospital Ramón y Cajal, Madrid, Spain); Gael Combarros Herman, Judit Tirado Muñoz (IMIM, Barcelona, Spain).

**Statistical analysis:** Robin Vernooij (Iberoamerican Cochrane Centre. IIBSant Pau, Barcelona, Spain); Victor Abraira (Unidad de Bioestadística Clínica. Hospital Universitario Ramón y Cajal. IRYCIS, Madrid, Spain).

**Co-investigators:**

### Barcelona, Spain

Virginia Becerra Bachito, Montserrat Ferrer Fores, Stefanie Schmidt, Olatz Garin, Yolanda Pardo (IMIM - Hospital del Mar Medical Research Institute-); Albert Frances (Hospital del Mar); Carola Orrego Villagran, Rosa Suñol (Instituto U. Avedis Donabedian); Dimelza Osorio, Gemma Sancho Pardo, Ignasi Bolívar, José Pablo Maroto, Mª Jesús Quintana, Martin Lorente, Cristina (Hospital de la Santa Creu i Sant Pau); Ferran Algaba, Palou Redorta, Salvador Esquena (Fundació Puigvert); Jordi Bachs (Fundació Privada Hospital de la Santa Creu i Sant Pau); Mª José Martínez Zapata (Iberoamerican Cochrane Centre. IIBSant Pau).

### Bilbao, Spain

Amaia Martínez Galarza, José Ignacio Pijoán Zubizarreta, Lorea Martínez (Hospital Universitario Cruces-BioCruces Health Research Institute).

### Tenerife, Spain

David Manuel Castro Diaz, Juan Manuel Ramos Goñi, Julio López Bastida (HTA Unit of the Canary Islands Health Service).

### Granada, Spain

Armando Suárez Pacheco, Cesar García López, Jose Manuel Cozar Olmo (Hospital Universitario Virgen de las Nieves); Carmen Martínez, Daysy Chang Chan, Mª Jose Sanchez Perez (Escuela Andaluza de Salud Pública).

### Madrid, Spain

Ana Isabel Díaz Moratinos, Angel Montero Luis, Asunción Hervás, Carmen Vallejo Ocaña, Costantino Varona, Javier Burgos, Javier Zamora, Jose Alfredo Polo Rubio, Luis López-Fando Lavalle, Miguel Angel Jiménez Cidre, Muriel Garcia, Alfonso, Nieves Plana Farras, Rosa Morera López, Sonsoles Sancho Garcia, Víctor Abraira, Victoria Gómez Dos Santos (Hospital Ramón y Cajal); Agustín Gómez de la Cámara, Javier de la Cruz, Juan Passas Martínez, Humberto García Muñoz, Mª Ángeles Cabeza Rodríguez (Hospital 12 de Octubre).

### San Sebastián, Spain

Irune Ruiz Díaz, José Ignacio Emparanza, Juan Pablo Sanz Jaka (Hospital Universitario Donostia).

### Valencia, Spain

Agustín LLopis González, María Morales (Universidad de Valencia); Carlos Camps, Cristina Caballero Díaz, Emilio Marqués Vidal, Francisco Sánchez Ballester, Joaquín Ulises Juan Escudero, Jorge Pastor Peidro, José López Torrecilla, Mª Macarena Ramos Campos, Miguel Martorell Cebollada (Consorcio Hospital General Universitario de Valencia).

## Abbreviations

PSA: Prostate-specific antigen
SD: Standard deviation
IQR: Interquartile range
BMI: Body mass index
OR: Odds ratio
ICC: Intra-cluster correlation coefficient
MOR: Median odds ratio
